# Clinical Presentation and Birth Outcomes Associated with Respiratory Syncytial Virus Infection in Pregnancy

**DOI:** 10.1371/journal.pone.0152015

**Published:** 2016-03-31

**Authors:** Helen Y. Chu, Joanne Katz, James Tielsch, Subarna K. Khatry, Laxman Shrestha, Steven C. LeClerq, Amalia Magaret, Jane Kuypers, Mark C. Steinhoff, Janet A. Englund

**Affiliations:** 1 Department of Medicine, University of Washington, Seattle, WA, 98102, United States of America; 2 Department of International Health, Johns Hopkins Bloomberg School of Public Health, Baltimore, MD, United States of America; 3 Department of Global Health, George Washington University, Washington, D.C., United States of America; 4 Nepal Nutrition Intervention Project-Sarlahi, Sarlahi, Nepal; 5 Department of Pediatrics and Child Health, Institute of Medicine, Tribhuvan University, Kathmandu, Nepal; 6 Department of Laboratory Medicine, University of Washington, Seattle, WA, 98102, United States of America; 7 Department of Biostatistics, University of Washington, Seattle, WA, 98102, United States of America; 8 Global Health Center, Cincinnati Children’s Hospital, Cincinnati, OH, 45229, United States of America; 9 Department of Infectious Diseases, Seattle Children’s Hospital, University of Washington, Seattle, WA, 98105, United States of America; University of North Carolina School of Medicine, UNITED STATES

## Abstract

**Background:**

Respiratory syncytial virus (RSV) is the most important cause of viral pneumonia in children worldwide. A maternal vaccine may protect both the mother and infant from RSV illness. The epidemiology and clinical presentation of RSV in pregnant and postpartum women is not well-described.

**Methods:**

Data were collected from a prospective, randomized trial of influenza immunization in pregnant women in rural southern Nepal. Women were enrolled in their second trimester of pregnancy and followed until six months postpartum. Active weekly home-based surveillance for febrile respiratory illness was performed. Mid-nasal swabs collected with episodes of respiratory illness were tested for RSV by real-time polymerase chain reaction.

**Results:**

RSV was detected in 14 (0.4%) illness episodes in 3693 women over 3554 person-years of surveillance from 2011–2014. RSV incidence was 3.9/1000 person-years overall, and 11.8/1000 person-years between September and December. Seven (50%) women sought care for RSV illness; none died. Of the 7 (50%) illness episodes during pregnancy, all had live births with 2 (29%) preterm births and a median birthweight of 3060 grams. This compares to 469 (13%) preterm births and a median birthweight of 2790 grams in women without RSV during pregnancy. Of the 7 mothers with postpartum RSV infection, RSV was detected in 4 (57%) of their infants.

**Conclusions:**

RSV was an uncommon cause of febrile respiratory illness in mothers during pregnancy in Nepal. These data will inform prevention and therapeutic strategies against RSV in resource-limited settings.

## Introduction

Respiratory syncytial virus (RSV) is the most important cause of acute lower respiratory infection (ALRI) in children worldwide [1]. RSV antibody protects young infants from severe ALRI. No RSV vaccines are currently licensed, though a RSV vaccine for use in pregnant women recently completed a phase II clinical trial in the United States [2, 3]. A RSV vaccine administered during pregnancy has the potential to protect the infant through transplacental antibody transfer and to decrease maternal RSV illness incidence and severity.

Maternal immunization against influenza, tetanus, and pertusiss has been shown to be safe, immunogenic, and efficacious in preventing disease in infants [2]. Influenza infection during pregnancy is associated with increased maternal and neonatal morbidity and mortality [4]. Maternal influenza vaccination is associated with decreased risk of adverse birth outcomes, such as low birthweight or small-for-gestational age infants [5–7]. RSV infection is associated with a similar disease burden to influenza in high-risk and elderly adults, and may be associated with adverse outcomes in pregnancy [8, 9]. The clinical presentation of RSV illness during pregnancy and the postpartum period and the effect of RSV infection on maternal, fetal, and infant outcomes are not well-described in large prospective studies.

The objective of this study was to describe the epidemiology and clinical presentation of respiratory syncytial virus infection using active home-based surveillance for respiratory illness in pregnant women in a prospective study.

## Materials and Methods

Two consecutive randomized placebo-controlled trials of maternal influenza immunization were conducted in two annual cohorts in Sarlahi District of southern Nepal from April 2011 to May 2014 (Clinicaltrials.gov identifier: NCT01034254) [10]. Women were enrolled in the second trimester of pregnancy and followed with weekly home-based visits until 180 days postpartum. Symptom information was captured by recall over the previous week at each visit. Maternal respiratory illness required reported or measured fever (>38°C) with at least one other symptom: cough, myalgia, sore throat or rhinorrhea. Infant respiratory illness was defined as having any of the following: fever, cough, wheeze, difficult or rapid breathing, or a draining ear. Participants who reported protocol-defined respiratory illness had mid-nasal swabs (NS) obtained by field workers during weekly visits. An RSV illness episode was defined as the presence of any symptoms occurring for a minimum of one day with a mid-nasal swab obtained and RSV detected on PCR assay, with a minimum of 7 symptom-free days separating illness episodes. Incidence of RSV was calculated using days of follow-up from enrollment until end of study or loss to follow-up.

Clinical and sociodemographic data were collected at enrollment and at weekly visits, and birthweight was measured at a postpartum home visit. Birthweight was included in the analysis if obtained within the first 72 hours after birth. Low birthweight was defined as weight < 2500 grams. Preterm birth was defined as birth at < 37 weeks completed gestation. Gestational age was calculated based on last menstrual period according to previously published methods [11]. Small-for-gestational age was defined according to the Alexander criteria [12]. A household was defined as the persons sharing a common cookstove. Household density was defined as the numbers of persons/room in the household. Statistical testing was performed using Wilcoxon rank-sum tests and Fisher’s exact tests to compare clinical and sociodemographic characteristics (SAS 9.3, Cary, NC).

Mid-nasal swabs were transported in a temperature-stable nucleic acid buffer (Primestore, Longhorn Diagnostics, Austin, TX) to the field laboratory, where they were aliquoted and stored at 4°Celsius until transport to the University of Washington Molecular Virology Laboratory in Seattle, WA, U.S.A [13]. RSV testing was performed using real-time reverse-transcriptase polymerase chain reaction (qRT-PCR) [13]. Specimens with RSV detected were additionally tested for quantitative viral load and subtype [14–16].

IRB approval for the study was obtained from the Johns Hopkins University Bloomberg School of Public Health, Seattle Children’s Hospital, Cincinnati Children’s Hospital, the Institute of Medicine at Tribhuvan University, and the Nepal Health Research Council. Oral consent was obtained from study participants due to low literacy rates in the population. This procedure was approved by the IRB.

## Results

**Fig 1:**
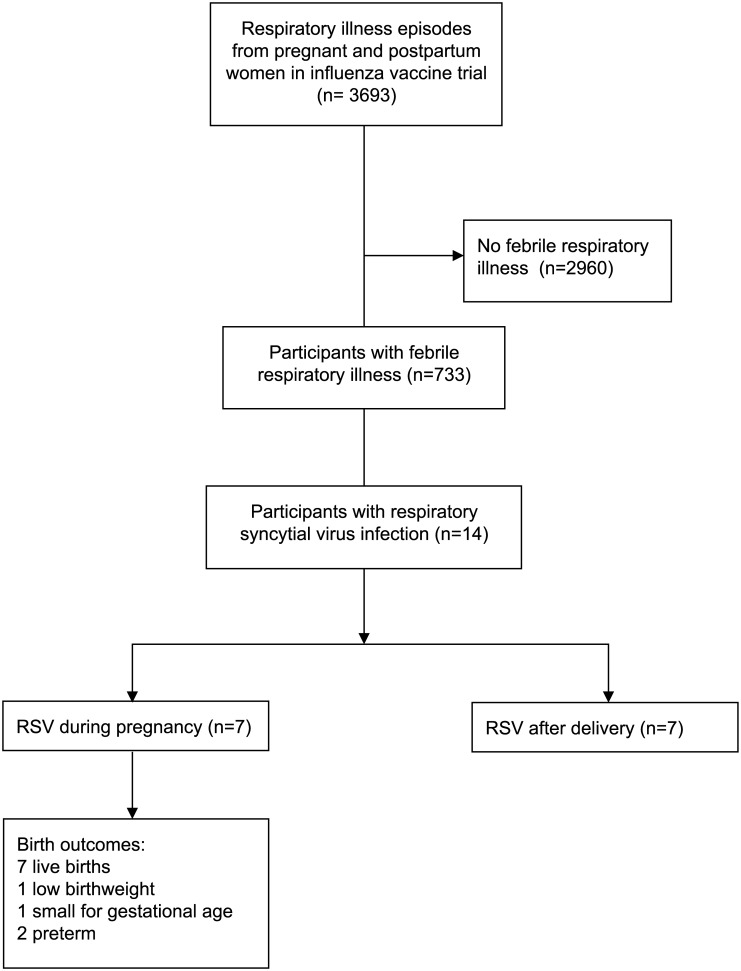
Flow diagram of study participants. Study participants were monitored with weekly active home-based surveillance for febrile respiratory illness over three years.

Altogether, 3693 pregnant women were enrolled and followed from April 2011 to May 2014; 944 nasal swabs were collected during 3554 person-years of follow-up while pregnant or postpartum, with a respiratory virus detected in 556 (59%) of these swabs. RSV was detected in nasal swabs collected from 14 (0.4%) of all women, which constitutes 2.0% of the 733 women with febrile respiratory illnesses (Fig 1). RSV was detected in women only between September and December during the three years of the study. The incidence of RSV was 3.9/1000 person years overall. A total of 1189 person-years of follow-up occurred between September and December; during this period, the incidence of RSV was 11.8/1000 person-years.

Overall, the median gestational age at enrollment was 17 weeks (range, 4–34). For mothers who had RSV, the median age at enrollment was 16 weeks, (range, 8–31) and for mothers without RSV, the median age at enrollment was 17 weeks (range, 4–34). Overall, 90% of moms were enrolled prior to 28 weeks gestation. Seven women had an RSV illness during pregnancy (median days before birth: 42 days, range, 13–201 days) and seven had RSV illness after delivery (median days after birth: 74, range 27–168). Baseline characteristics of women with and without RSV infection are shown in Table 1. Mothers with RSV report fewer median years of education than those without (0 vs. 5 years; P = 0.04). No differences were observed in number of other children in the household (P = 0.80), use of an open indoor cookstove (P = 0.09), or presence of household members who smoked (P = 0.73).

**Table 1:** Comparison of sociodemographic and clinical characteristics of women with and without RSV infection.

Characteristic, median (range) or N(%)	RSV-positive, (n = 14)	RSV-negative (n = 3679)	P-value^a^
Maternal age at enrollment	24 (17, 31)	23 (13, 45)	0.99
Maternal smoking	0 (0)	111/3668 (3)	0.99
Primiparity	6 (43)	1542/3672 (42)	0.99
Maternal years of education	0 (0, 18)	5 (0, 18)	0.040
No. household members	7 (3, 12)	7 (1, 30)	0.55
No. children < 15 years	1 (0, 5)	2 (0, 13)	0.80
No. children < 5 years	1 (0, 2)	1 (0, 7)	0.69
No. school going children	1 (0,3)	1 (0, 10)	0.88
Household density	3 (1, 12)	3 (0, 20)	0.61
Household latrine	7 (50)	1740 (49)	0.99
Household smoking	5/13 (38%)	1505/3476 (43%)	0.73
Traditional open cookstove use	14 (100)	2881/3576 (81)	0.087
Vaiysha caste	9 (64)	1954/3583 (55)	0.60

^a^Wilcoxon test used for continuous measures and Fisher’s exact tests for dichotomous measures

### Clinical and Virologic Characteristics of RSV Illness Episodes

**Fig 2:**
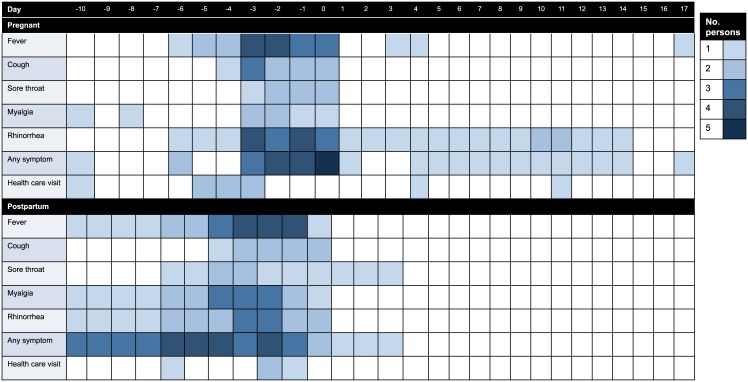
Heatmap of symptoms in women with respiratory syncytial virus infection. Heatmap with symptoms in women with respiratory syncytial virus infection during pregnancy (top) and after delivery (bottom) over the duration of the illness episodes. The color intensity of the box represents the number of participants with symptoms, with each box representing one day.

RSV illness in pregnant and postpartum women was associated with a median of 2 days of fever (range, 1–7), 2 days of myalgia (range, 0–5), 1 day of cough (range, 0–5), 2 days of rhinorrhea (range, 0–18), and 0 days of sore throat (range, 0–10), with a median of 4 days of any symptoms (range, 2–18) (Table 2; Fig 2). Seven (50%) mothers sought care at local health care facilities for their RSV illness. Of these, two visited a medicine shop and five saw a practitioner. No mothers with RSV infection died.

**Table 2:** Illness episode characteristics of mothers with RSV infection during pregnancy and the postpartum period.

Illness characteristics	During pregnancy (n = 7)	Post-partum (n = 7)	P-value^a^
RSV viral load (log_10_ copies/mL)	4.0 (2.3, 8.5)	4.9 (3.3, 8.1)	0.35
RSV subtype A	4/5 (80%)	5 (71%)	0.99
Days of any symptoms	3 (2, 18)	4 (2, 10)	0.44
Fever	2 (1, 7)	4 (1, 5)	0.89
Cough	2 (0, 3)	0 (0, 5)	0.45
Myalgia	0 (0, 2)	2 (0, 5)	0.20
Rhinorrhea	2 (0, 18)	2 (0, 7)	0.60
Sore throat	0 (0, 3)	0 (0, 10)	0.71
Visit for care	4 (57)	3 (43)	1.00

^a^Wilcoxon test for continuous measures and Fisher’s exact test for categorical measures.

The median RSV viral load was 4.1 log_10_ copies/mL (range, 2.3–8.5). RSV subtype A was detected in 9 (75%) of 12 samples with subtyping performed. An additional respiratory virus was detected in five illness episodes, including coronavirus (n = 1; 20%), rhinovirus (n = 2; 40%), parainfluenza 2 (n = 1; 20%), human metapneumovirus (n = 1; 20%), and bocavirus (n = 1; 40%).

### Effect of RSV infection in Pregnancy on Birth & Infant Outcomes

All seven women with RSV illness during pregnancy delivered live born infants (Table 3). Two (29%) infants were born prematurely at 34 and 36 weeks gestation to mothers who had RSV six and two weeks before birth, respectively. Of the five infants with known birthweights, 1 (20%) was low birthweight and 1 (20%) was small for gestational age. Among infants born to women without RSV during pregnancy, 678 (25%) of 2736 infants with weights measured within 72 hours of delivery were low birthweight. In addition, 469 (13%) of 3612 infants were premature and 63 (2%) of 3719 infants were stillbirths. Growth data was available for 5 (63%) infants born to pregnant women with RSV; median six month Z-score for weight was -0.8 (IQR, -3.0–0.7) and six month Z-score for length was -0.5 (IQR, -2.1–0.4). For infants born to mothers without RSV during pregnancy, the median six-month Z-score for weight was -1.0 (IQR, -8.5–3.7) and six-month Z-score for length was -1.1 (IQR, -12.0–3.7). With the small number of infants born to mothers having had RSV during pregnancy, no statistical testing was done to compare these characteristics.

**Table 3:** Birth outcomes in women with and without RSV infection during pregnancy.

Characteristic, median (range) or N(%)	RSV during pregnancy (n = 7)	No RSV during pregnancy (n = 3686)
Infant birth weight (g)^a^	3060 (2380, 3210)	2790 (820, 4800)
Infant length (cm)^b^	47 (45, 49)	48 (32, 67)
Infant gest age (weeks)^c^	37 (34, 42)	40 (28, 42)
Stillbirth	0 (0)	63 (2)
Low birthweight^a^	1 (20)	678 (25)
Preterm^c^	2 (29)	469 (13)
Small-for-gestational-age	1 (20)	1495 (55)
Six month weight (g)^d^	7180 (5070, 8530)	6740 (1540, 11720)
Six month length (cm)^d^	65 (61, 68)	64 (41, 76)
Six month weight Z score	-0.8 (-3.0, 0.7)	-1.0 (-8.5, 3.7)
Six month length Z score	-0.5 (-2.1, 0.4)	-1.1 (-12.0, 3.7)

^a^Available for 5 infants born to women with RSV and 2736 live born infants born to non-RSV mothers, infant weight included only if measured within 72 hours after birth

^b^Available for 7 infants born to women with RSV and 3391 live born infants born to non-RSV mothers.

^c^Available for 7 infants born to women with RSV and 3612 infants born to non-RSV mothers.

^d^Available for 5 infants born to women with RSV and 2624 infants born to non-RSV mothers.

### Postpartum RSV Infection within Mother-Infant Pairs

Among the 7 mothers with RSV in the six months following birth, 4 of their infants also had RSV. Two infant-mother pairs had RSV detected on the same day. For two additional pairs, the infant and mother had RSV four weeks apart. Of these two, in one pair, the infant had RSV detected four weeks following maternal RSV detection and in the other, the mother had RSV detected four weeks following infant RSV detection. All 3 pairs with RSV typing available from both the mother and infant had the same subtype (2 subtype A; 1 subtype B). The median differences in RSV viral load between mothers and infants was 1.9 log_10_ copies/mL (range, 1.5–3.5).

## Discussion

Active home-based viral surveillance in rural Nepal was utilized to determine the incidence and clinical presentation of febrile RSV disease in pregnant and postpartum women. RSV was an uncommon cause of febrile respiratory illness in pregnant women, and did not result in any hospitalizations or deaths in this population.

Maternal vaccination is recommended in many countries to protect women and their infants against influenza, tetanus, and pertussis [2]. Influenza infection during pregnancy may be associated with increased risk of maternal mortality, as well as low birthweight and small-for-gestational age infants [6]. The mechanism for this is unknown, but may be related to physiologic changes related to pregnancy, decreased cardiopulmonary reserve leading to increased severity of illness, and modulation of the systemic immunologic response to infection [17]. Because of the increased risk of maternal mortality and adverse birth outcomes associated with influenza during pregnancy, the Centers for Disease Control in the United States recommends influenza vaccine be administered at any time during pregnancy [18]. In contrast, pertussis in adults has a relatively milder course compared to the high morbidity and mortality due to pertussis infection in infants. Therefore, pertussis vaccines in pregnancy are administered in the third trimester to maximize transplacental antibody transfer to the infant [19]. RSV transplacental antibody transfer is efficient in mother-infant pairs in South Asia, the United States, and the Gambia [20]. Higher cord blood RSV antibody titers in Bangladesh are associated with decreased risk of serologic infection in infants [21]. Because febrile RSV illness was rare and relatively mild in pregnancy, a potential vaccination strategy would be similar to the pertussis strategy, with prioritization of infant protection from severe disease. Vaccination during the third trimester when antibodies are most likely to be transferred to the infant would maximize protection of the infant in the early postpartum period.

Assessment of risks of RSV infection in women becomes more important as maternal immunization with RSV subunit vaccines is considered as an approach to prevent symptomatic lower respiratory tract disease in both mothers and infants [22]. A phase II trial of a maternal RSV vaccine candidate was recently found to be safe and immunogenic in non-pregnant women of childbearing age [3]. As trials expand to resource-limited settings, it will be important to know baseline incidence rates of RSV infection and adverse birth outcomes in the study population. Previous estimates of RSV incidence in adults in low and middle-income countries have been calculated using outpatient and hospital-based surveillance with estimates ranging from 0.13 to 4.4/1000 person-years in Kenya, Egypt, and Thailand [23–25]. In the United States, the incidence of RSV in healthy elderly adults was 8.7/1000 person-years; however, in this study, surveillance was only performed between November and April during periods of RSV circulation [8]. The incidence rates of 11.8/1000 person-years during RSV season in this study are comparable to prior studies. The seasonality of RSV in southern Nepal is similar to that observed in previous studies performed in northern India and other regions of Nepal, with a clear peak during the months of September through November [26, 27]. This is in contrast to influenza, which has a less defined season in tropical as compared to temperate regions of the world [28–30].

RSV was an uncommon cause of febrile respiratory illness in the participants in our study. The median duration of fever was only two days, and total symptom duration was four days. No participants in this study were hospitalized or died due to RSV; this is in contrast to a case report of pregnant women with RSV at an academic medical center in the United States where two of three patients were mechanically ventilated [9]. In contrast to this case report, our study used active home-based surveillance in rural south Asia, a population with low rates of obesity and other risk factors that may increase risk for respiratory failure.

RSV was also detected in a substantial proportion of infants of mothers with postpartum RSV illness, with evidence of the same subtype in mother-infant pairs. This agrees with studies showing mothers as a potential source of RSV introduction to infants in households in rural Kenya [31]. Maternal vaccination may potentially reduce the spread of RSV within mother-infant pairs and other household members if administered close to the time of delivery.

Limitations of our study include lack of collection of samples in mothers whose respiratory symptoms did not include fever, or in mothers without any symptoms. In family studies, RSV is associated with fever in only 5–27% of illness episodes [32]. These studies suggest the incidence of RSV-associated afebrile respiratory illness could be several-fold higher than found in our study, and potentially account for significant numbers of days with symptoms and health care seeking. Febrile respiratory illness due to RSV in our population did result in a median of four days of symptoms and half of the mothers seeking care for their illness. This is in a region of the world where access to health care is limited by poverty, distance, and gender inequality. Studies in healthy military recruits have found that RSV accounts for 11% of clinically significant respiratory illness in this population, and accounted for one day of missed work in 94% of the cases [33]. An additional limitation was our inability to describe the effect of RSV infection on birthweight or other birth outcomes due to the small numbers of pregnant or postpartum women with febrile RSV illness. It is possible that milder cases of RSV disease without fever can have adverse effects on maternal and infant outcomes, and we were unable to assess this in our study. We did find, however, no mortality or hospitalization due to RSV in this population.

## Conclusions

In conclusion, symptomatic febrile RSV illness during pregnancy and after delivery was rare among mothers in south Asia. These data will inform prevention and therapeutic strategies against RSV in resource-limited settings.
